# Cost‐effectiveness analysis of pharmacogenetic‐guided antiseizure medication therapy based on the risk of HLA‐A*31:01 allele variants in Japan

**DOI:** 10.1002/pcn5.70249

**Published:** 2025-11-20

**Authors:** Yasushi Maruyama, Hiroki Kimura, Kohei Ninomiya, Takuji Nishida, Shiori Ogawa, Masashi Ikeda

**Affiliations:** ^1^ Department of Psychiatry Nagoya University Graduate School of Medicine Nagoya Aichi Japan; ^2^ National Epilepsy Center, NHO Shizuoka Institute of Epilepsy and Neurological Disorders Shizuoka Japan; ^3^ Department of Psychiatry Fujita Health University School of Medicine Toyoake Aichi Japan; ^4^ Research Center of Health, Physical Fitness and Sports Nagoya University Nagoya Aichi Japan

**Keywords:** carbamazepine, cutaneous adverse drug reactions, epilepsy, pharmacogenetics, public health

## Abstract

**Aim:**

Carbamazepine (CBZ) remains a common antiseizure medication for focal epilepsy but carries the risk of cutaneous adverse drug reactions (cADRs), including Stevens–Johnson syndrome and toxic epidermal necrolysis. The HLA‐A*31:01 allele is associated with increased risk of CBZ‐induced cADRs, with notably high prevalence (8.4%) in the Japanese population. While genetic screening before CBZ treatment has proven cost‐effective in other populations, its economic value in Japan remains unexplored.

**Methods:**

We conducted a cost‐effectiveness analysis comparing two strategies for newly diagnosed focal epilepsy treatment: (1) screening HLA‐A*31:01 before CBZ treatment, with CBZ for negative results and levetiracetam for positive results, and (2) CBZ treatment without screening. Effectiveness was measured in quality‐adjusted life years (QALYs) based on utility values scored from 0 (*death*) to 1 (*perfect health*). We developed a decision tree model based on a Markov model with monthly cycles and a 20‐year follow‐up, analyzed from the Japanese healthcare perspective. We utilized clinical trial data and regional cost estimates to conduct base‐case and sensitivity analyses. The willingness‐to‐pay (WTP) threshold was set at 33,784 USD.

**Results:**

The incremental cost‐effectiveness ratio was 6956 USD/QALY, below the WTP threshold of 33,784 USD, indicating high cost‐effectiveness. Probabilistic sensitivity analyses revealed that the probability of the HLA screening strategy being cost‐effective was 97.0%, confirming stable cost‐effectiveness.

**Conclusion:**

HLA‐A*31:01 screening before CBZ treatment is cost‐effective in the Japanese population. These findings support incorporating genetic screening into Japan's healthcare coverage system to enable more personalized medicine approaches in epilepsy treatment.

## Graphical abstract

This study was the first to evaluate the cost‐effectiveness of genetic testing (HLA‐A*31:01) before prescribing carbamazepine, an epilepsy medication, to prevent severe skin reactions in Japanese patients. The HLA screening strategy, which uses alternative medications for high‐risk patients, was found to be cost‐effective compared to the conventional approach of treating all patients with the same medication without testing.

## INTRODUCTION

Carbamazepine (CBZ) is a widely used antiseizure medication, initially developed for the treatment of focal epilepsy and later approved for various neurological and psychiatric conditions.

Recent guidelines, such as the National Institute for Health and Care Excellence guidelines (NICE),^1^ have shifted away from recommending CBZ as first‐line treatment for newly diagnosed epilepsy. However, CBZ remains widely prescribed in clinical practice, particularly in resource‐limited settings and for cost considerations. Notably, the Japanese Society of Neurology Guidelines for the Treatment of Epilepsy 2018 continues to recommend CBZ as a first‐line treatment for focal epilepsy, reflecting regional differences in treatment recommendations. In Japan, CBZ continues to be commonly used due to its established efficacy profile and lower cost compared to newer antiseizure medications. However, CBZ is associated with a significant risk of cutaneous adverse drug reactions (cADRs). These range from mild rashes to severe, life‐threatening conditions such as Stevens–Johnson syndrome (SJS) and toxic epidermal necrolysis (TEN).

Pharmacogenomic studies have identified several HLA alleles as risk factors for developing CBZ‐induced cADRs in different populations.^2^, ^3^, ^4^, ^5^ One of these risk alleles, HLA‐B*15:02, has been identified as a significant risk factor for CBZ‐induced severe cADRs in various Asian populations, especially the Han Chinese, Thai, and Malaysian populations, which have high HLA‐B*15:02 allele frequencies (>10% in some populations) according to the Allele Frequency Net Database (https://www.allelefrequencies.net/).^6^, ^7^, ^8^, ^9^ In contrast, the Japanese population has a much lower frequency of this allele (0.03%–0.1%), making HLA‐B*15:02 screening less relevant for CBZ safety in Japan. Cost‐effectiveness analyses of HLA‐B*15:02 screening prior to CBZ administration have been evaluated across various Asian populations,^10^, ^11^, ^12^ resulting in the recommendation of HLA‐B*15:02 screening before CBZ treatment^13^ by the US Food and Drug Administration and the proper authorities of several Asian countries.

Another risk allele, HLA‐A*31:01, is also associated with an increased risk of CBZ‐induced cADRs,^3^, ^14^ with an odds ratio of 10.8.^3^ The prevalence of HLA‐A*31:01 in the Japanese population is 8.4% (Allele Frequency Net Database; https://www.allelefrequencies.net/), which is substantially higher than that in other populations (e.g., 1.8% in the Han Chinese,^3^ 2%–5% in Northern Europeans^4^). However, while the Clinical Pharmacogenetics Implementation Consortium guidelines recommend screening for high‐risk HLA alleles, including HLA‐A*31:01,^15^ before CBZ administration, HLA allele screening has no recommendation guidance in Japan because of the unique circumstances of the medical insurance system. Japan's universal health insurance system implements a unique cost‐sharing model where patients typically bear 30% of medical expenses, which can be reduced to 10% for patients with epilepsy through the Medical Payment for Services and Supports for Persons with Disabilities system. This differs significantly from other healthcare systems: the US relies primarily on private insurance with variable coverage, while European countries like the UK provide comprehensive coverage through national health services. The strict coverage criteria in Japan's system particularly affect genetic testing, where HLA allele testing remains limited to specific conditions like hematopoietic stem cell transplantation. This regulatory environment creates unique economic barriers to implementing HLA‐A*31:01 screening despite its clinical benefits. As a result, HLA allele testing for pre‐prescription screening of medications such as CBZ or as a diagnostic aid for diseases such as narcolepsy and Behçet's disease is not covered by insurance, and the full cost must be borne by either the patient or healthcare institution. Furthermore, awareness about the importance of HLA screening among healthcare professionals remains insufficient, which hinders its widespread adoption.

Considering Japan's unique medical system and genetic population, it is important to evaluate the cost‐effectiveness of screening HLA‐A31:01 before CBZ treatment. Therefore, this study aimed to clarify the economic validity of HLA‐A31:01 screening, considering both the characteristics of Japan's universal healthcare system and the genetic risk factors specific to the Japanese population.

## METHODS

### Cost‐effectiveness analysis methodology

Cost‐effectiveness analysis serves as a systematic approach for evaluating healthcare interventions by comparing their economic burden and clinical benefits to inform resource allocation decisions. The primary metric is the incremental cost‐effectiveness ratio (ICER), calculated as the difference in costs divided by the difference in health outcomes, quantified in quality‐adjusted life years (QALYs).

Multiple methodological approaches exist for conducting these analyses, including trial‐based evaluations performed alongside clinical trials and model‐based evaluations using mathematical frameworks to simulate patient outcomes. In this investigation, we implemented a model‐based strategy combining decision tree and Markov model structures to capture treatment decisions and long‐term health state transitions.

Economic assessments incorporated direct medical expenditures, including treatment costs and medications. Clinical effectiveness was quantified using utility values from validated quality of life instruments such as EQ‐5D, which characterize health states on a scale from 0 (death) to 1 (perfect health). These utility values are combined with time in each health state to calculate QALYs.

Sensitivity analyses, including one‐way parameter variation and probabilistic Monte Carlo simulations with thousands of iterations, were conducted to evaluate result robustness under uncertainty. The calculated ICER was compared against willingness‐to‐pay thresholds (WTP), with interventions below these thresholds considered cost‐effective.

### Model structure

We compared two treatment strategies for newly diagnosed focal epilepsy:

(1) CBZ with an HLA‐A*31:01 screening strategy (HLA‐A*31:01 screening followed by CBZ for negative results and levetiracetam (LEV) for positive results).
(2) CBZ without an HLA‐A*31:01 screening strategy (initiating treatment with CBZ without screening for HLA‐A*31:01).

**Figure 1:**
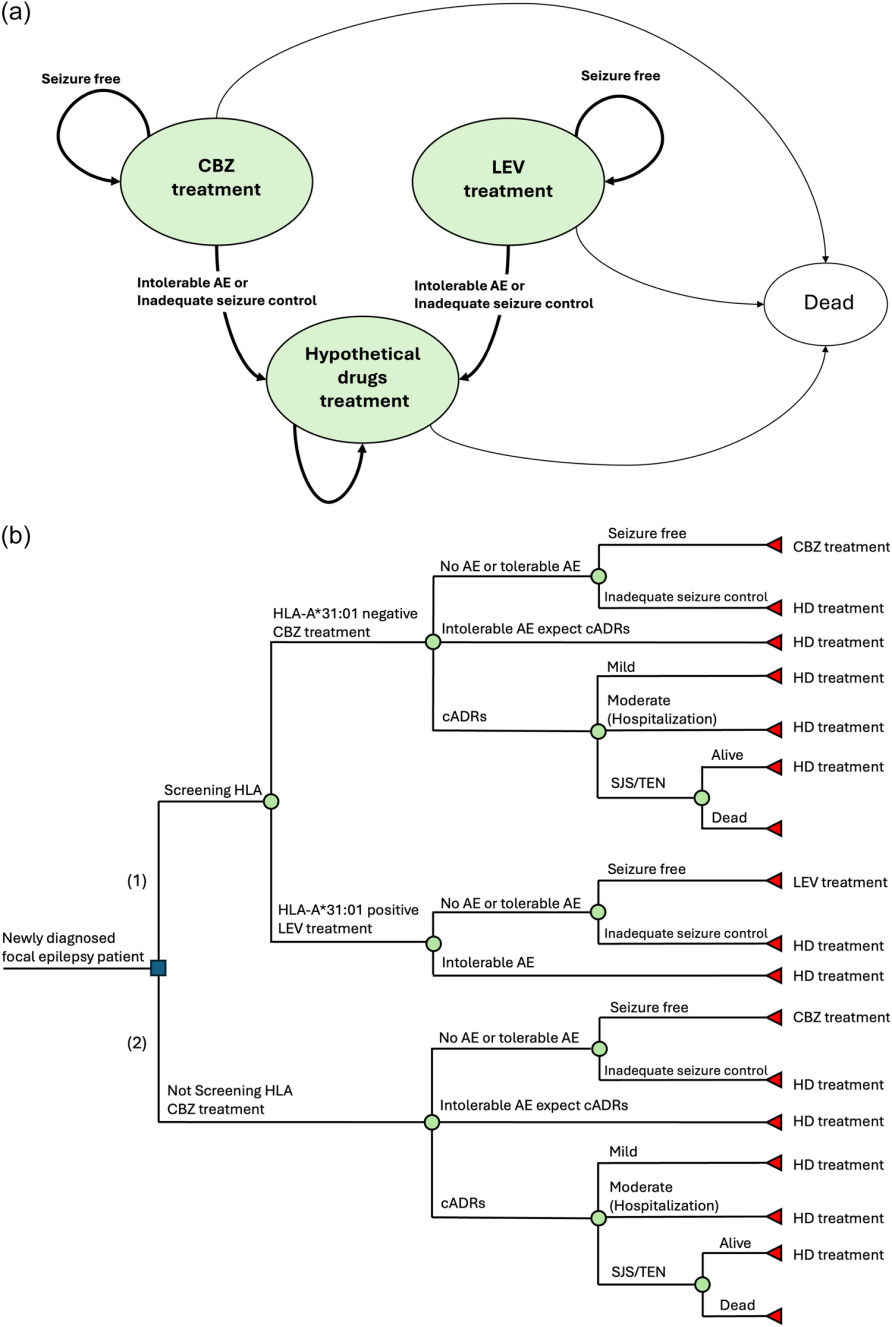
Model structure. (a) Markov model in this analysis: This model represents long‐term disease progression, showing transitions between health states over 20 years. Patients can have seizure‐free status, inadequate seizure control, or die. They may receive CBZ, LEV, or hypothetical drugs (HD) based on treatment response and adverse effects. (b) Decision tree model: This model compares two strategies. Strategy (1): CBZ with HLA‐A*31:01 screening, where HLA‐A*31:01‐positive patients receive LEV treatment, and HLA‐A*31:01‐negative patients receive CBZ treatment. Strategy (2): CBZ without HLA‐A*31:01 screening, where all patients receive CBZ treatment regardless of HLA status. AE, adverse effect; cADRs, cutaneous adverse drug reactions; CBZ, carbamazepine; HD, hypothetical drugs; LEV, levetiracetam; SJS, Stevens–Johnson syndrome; TEN, toxic epidermal necrolysis.

We developed a decision tree model based on a Markov model using TreeAge Pro Healthcare 2024 (TreeAge Software, Williamstown, MA, USA) to assess the cost‐effectiveness of different treatments for newly diagnosed focal epilepsy (Figure 1). This study followed the Consolidated Health Economic Evaluation Reporting Standards checklist^16^ to ensure comprehensive and transparent reporting.

Our model consists of two integrated components (Figure 1). Figure 1a (Markov model) represents the long‐term disease progression of epilepsy patients over 20 years, showing transitions between defined health states using monthly cycles with age‐adjusted mortality rates and a standardized mortality ratio of 1.42 for epilepsy patients.^17^ Health states were defined as follows: “Seizure Free” represents patients with no seizures for 12 consecutive months while on treatment, and “Inadequate seizure control” represents patients who continue to experience seizures despite treatment. Within this disease framework, patients can receive CBZ, LEV, or hypothetical drugs (HD) and transition to HD treatment when experiencing inadequate seizure control or intolerable adverse effects. Figure 1b (decision tree) compares the two treatment strategies using the disease progression framework from Figure 1a. The decision tree shows initial treatment allocation based on HLA screening results: HLA‐negative patients receive CBZ, HLA‐positive patients receive LEV, while patients in the no‐screening strategy receive CBZ regardless of HLA status. The model represented a group of Japanese patients with an average age of 40 years at epilepsy onset, based on the observed average onset age of 35–42 years.^18^

The model used monthly cycles to reflect changes in epilepsy management over time, and the follow‐up period was 20 years. All costs were standardized to USD using exchange rates as of October 2024 (1 USD = 148 JPY) to facilitate direct comparison across healthcare systems. The WTP thresholds were similarly converted to USD, ensuring that our cost‐effectiveness conclusions remain robust against exchange rate fluctuations since both costs and thresholds are expressed in the same currency. We applied a 2% annual discount rate to both costs and effects.^19^

To assess cost‐effectiveness, we calculated the ICER by comparing differences in costs and QALYs between strategies.

$\mathrm{ICER}=\;\frac{{\mathrm{Costs}}_{\mathrm{comparator\_strategy}}-{\mathrm{Costs}}_{\mathrm{baseline\_strategy}}}{{\mathrm{QALYs}}_{\mathrm{comparatpr\_strategy}}-{\mathrm{QALYs}}_{\mathrm{baseline\_strategy}}}$

The willingness‐to‐pay (WTP) threshold of Japan was set at 33,784 USD (5,000,000 JPY), consistent with health technology assessment standards.

### HLA‐A*3101 allele screening

Our HLA‐A*31:01 allele screening model was based on data specific to the Japanese population. The cost of each HLA‐A*31:01 screening test was set at 14,850 JPY (about 100 USD) based on current pricing from the Japan HLA Laboratory (https://hla.or.jp/) for clinical HLA typing services. We assumed that the HLA genotyping test itself was perfectly accurate (100% accuracy) in determining the presence or absence of the HLA‐A*31:01 allele.

### Epilepsy treatment

LEV was selected as the comparator drug based on several factors: (1) LEV is recommended as one of the first‐line treatment options for focal epilepsy alongside CBZ in the Japanese Society of Neurology Guidelines for the Treatment of Epilepsy 2018, (2) it has a well‐established safety profile that differs from CBZ, particularly regarding cutaneous adverse reactions, and (3) it is widely available and covered under Japanese health insurance, making it a realistic alternative in clinical practice.

Drug costs were calculated based on the 2024 Japanese drug price standard established by the Ministry of Health, Labour and Welfare, using generic formulations as the lowest price options. CBZ costs were derived from the standard maintenance dose of 600 mg/day (7.03 USD monthly), while LEV costs were based on the standard dose of 2000 mg/day (37.8 USD monthly). For alternative medications, we used hypothetical drugs representing the weighted average cost of second‐line antiseizure medications recommended by NICE guidelines for focal epilepsy, including lamotrigine, zonisamide, lacosamide, perampanel, and topiramate.

A comparative study of CBZ and LEV by Brodie et al. showed 12‐month seizure suppression rates of 58.5% and 56.6%, respectively,^20^ and in the present analysis model, the 12‐month seizure suppression rate for either drug was set at 60% for model simplicity and to focus the analysis specifically on the effects of HLA screening.

The annual dropout rates of 19.2% for CBZ and 14.4% for LEV were converted to monthly dropout probabilities using the standard formula: monthly probability = 1 − (1 − annual rate)^(1/12), yielding monthly dropout probabilities of 1.76% for CBZ and 1.28% for LEV.^20^ For long‐term projections, we assumed no further treatment discontinuations after the first 12 months for patients who remained on therapy. This assumption is based on clinical evidence that most treatment‐related discontinuations due to intolerable adverse effects occur within the first year of antiseizure medication therapy, and patients who tolerate treatment through this period typically continue long‐term without discontinuation due to adverse effects.

Health states were defined based on seizure control outcomes: patients achieving seizure freedom for 12 consecutive months were assigned a utility value of 0.94, while those with inadequate seizure control despite treatment were assigned a utility value of 0.86. These utility values were derived from EQ‐5D measurements in epilepsy patients.

### Cutaneous adverse drug reactions (cADRs)

In the Japanese population, the incidence of CBZ‐induced cADRs is reported to be 5.1%, of which 17.4% are moderate. Moderate cADRs were defined as cases requiring hospitalization but not reaching the severity of SJS/TEN, while mild cADRs were treated in outpatient settings. When HLA‐A*31:01 is used as a predictive test for identifying patients who will develop cADRs, it demonstrates a sensitivity of 60.7% and a specificity of 87.5%.^14^ Using Bayes’ theorem, it is possible to predict the incidence of cADRs in HLA‐A*31:01‐negative individuals. Based on previous prospective studies, the estimated incidence of cADRs in HLA‐A*31:01‐negative individuals is 2.2%. The incidence of CBZ‐induced SJS/TEN in the Japanese population is 0.23%.^14^ In HLA‐A*31:01‐positive patients, the relative hazard for SJS/TEN is 33.9 compared with HLA‐A*31:01‐negative individuals.^3^ Based on Bayes’ theorem, the incidence of SJS/TEN in HLA‐A*31:01‐negative individuals is 0.07%.

The utility values for cADRs are based on existing utility measurements from studies on burns and psoriasis.^21^, ^22^ Hospital admission costs in cADRs are determined in accordance with the Diagnosis Procedure Combination system based on the medical fees point system in Japan (2024).

### Sensitivity analysis

To address parameter uncertainty and validate the robustness of our findings, we employed both probabilistic sensitivity and one‐way sensitivity analyses. The probabilistic sensitivity analysis utilizes Monte Carlo simulation methodology, where model parameters are varied according to their respective probability distributions across 100,000 iterations to generate 95% confidence intervals for both cost and QALY outcomes. Additionally, we conducted one‐way sensitivity analyses by systematically varying each key model parameter individually within its plausible range to assess the impact on model results and identify influential variables.

Parameter ranges for sensitivity analysis were determined using ±2 standard deviations when literature‐based CIs were available, –50% to +200% variation around base‐case values for cost parameters, and clinically plausible ranges for probability parameters. When data from the literature were insufficient, we applied conservative ranges based on standard health economic modeling conventions and expert judgment. All parameter ranges are detailed in Table 1.

**Table 1:** Input parameters for the cost‐effectiveness^1^ analysis.

	Distribution	Mean (baseline)	SD	Lower range	Upper range	Reference
Cost (USD)						
Monthly cost of CBZ	Gamma	$7.03	1.4	$3.5	$14	The 2024 Japanese drug price standard was established by the Ministry of Health, Labour and Welfare
Monthly cost of LEV	Gamma	$37.8	7.6	$19	$76	
Monthly cost of hypothetical drugs	Gamma	$44.4	8.9	$22	$89	
Monthly cost of treatment for mild cADRs	Gamma	$135	27	$68	$270	Estimated value from the medical fees point system (2024)
Monthly cost of moderate cADRs	Gamma	$3,380	680	$1700	$6800	
Monthly cost of SJS/TEN	Gamma	$7,610	1500	$3800	$15,000	
Cost of HLA test	Gamma	$100	20	$50	$200	https://hla.or.jp/
Utility						
Utility of seizure‐free	Normal	0.94	0.03	0.88	0.99	Selai et al. 2005^25^
Utility of inadequate seizure control	Normal	0.86	0.03	0.81	0.91	Selai et al. 2005^25^
Utility of mild cADRs	Normal	0.82	0.05	0.7	0.99	Yang et al. 2015^22^
Utility of moderate cADRs	Normal	0.55	0.05	0.30	0.80	Intermediate value (between mild cADRs and SJS/TEN)
Utility of SJS/TEN	Normal	0.31	0.05	0.10	0.50	Sánchez et al. 2007^21^ Dong et al. 2012^11^
Probability						
Prevalence of HLA‐A*31:01 in the Japanese population	Beta	0.084	0.002	0.04	0.17	Allele Frequency Net Database (https://www.allelefrequencies.net/)
Probability of 12 months seizure‐free for CBZ treatment	Beta	0.585	0.033	0.5	0.7	Brodie et al. 2007^20^
Probability of 12 months seizure‐free for CBZ treatment	Beta	0.566	0.033	0.5	0.7	Brodie et al. 2007^20^
Probability of 12 months intolerable adverse effect of CBZ	Beta	0.192	0.023	0.1	0.25	Brodie et al. 2007^20^
Probability of 12 months intolerable adverse effect before LEV	Beta	0.144	0.021	0.1	0.2	Brodie et al. 2007^20^
Probability of all cADRs after CBZ in the Japanese population	Beta	0.051	0.010	0.02	0.1	Mushiroda et al. 2018^14^
Probability of all cADRs after CBZ in the HLA‐A*31:01‐negative population	Beta	0.022	0.0044	0.0	0.045	(Derived from Bayes’ theorem)
Probability of SJS/TEN after CBZ in the Japanese population	Beta	0.0023	0.00069	0.0	0.010	Mushiroda et al. 2018^14^
Probability of SJS/TEN after CBZ in the HLA‐A*31:01‐negative population	Beta	0.0007	0.00028	0.0	0.0015	(Derived from Bayes’ theorem)
Mortality of SJS/TEN	Beta	0.22	0.044	0.05	0.3	Miliszewski et al. 2016^26^
Others						
SMR of PWEs	‐	1.42	‐	1.00	3.00	Mohanraj et al. 2006^17^
Annual discount rate	‐	2%	‐	0%	5%	

*Note*: 1 USD = 148 JPY.

Abbreviations: cADRs, cutaneous adverse drug reactions; CBZ, carbamazepine; LEV, levetiracetam; PWEs, patients with epilepsy; SD, standard deviation; SJS, Stevens–Johnson syndrome; SMR, standardized mortality ratio; TEN, toxic epidermal necrolysis.

## RESULTS

### Base‐case analysis

The parameters for this analysis are presented in Table 1. In the primary base‐case analysis, we used baseline parameters and evaluated two strategies: CBZ with HLA‐A*31:01 screening and CBZ without HLA‐A*31:01 screening. For the screening strategy, the average cost was 5366.6 USD and the average QALYs were 14.6452, while for the no‐screening strategy, the average cost was 5455.1 USD and the average QALYs were 14.6325. The incremental cost and QALY gains were 88.6 USD and 0.0127, respectively, resulting in an ICER of 6956 USD/QALY gained. This ICER was well below the WTP threshold of 33,784 USD/QALY; therefore, the CBZ with HLA‐A*31:01 screening strategy was determined to be cost‐effective (Table 2).

**Table 2:** Cost‐effectiveness of the primary base case.

Strategy	Cost (USD)	Incremental cost (USD)	Effect (QALY)	Incremental effect (QALY)	ICER (USD/QALY)
CBZ without HLA screening	5455.2	‐	14.6325	‐	‐
CBZ with HLA screening	5366.6	88.6	14.6452	0.0127	6956

### Sensitivity analyses

**Figure 2:**
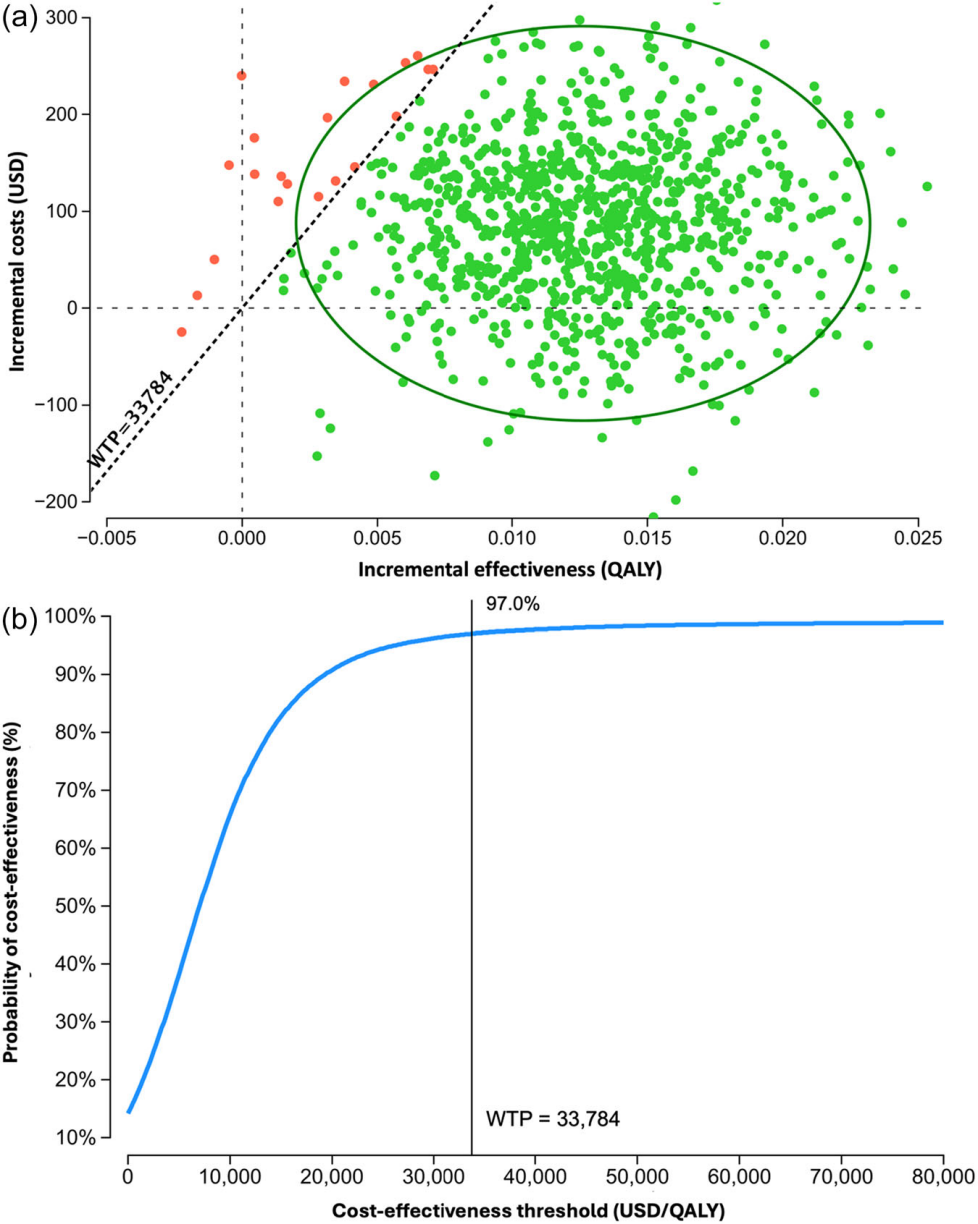
Probabilistic Sensitivity Analysis. (a) Scatterplot for incremental costs and effectiveness. Each plot represents the result of the Monte Carlo simulation. (b) Cost‐effectiveness acceptability curve. The curve represents the probability that each of the two strategies exceeds the WTP threshold. CBZ, carbamazepine; LEV, levetiracetam; WTP, willingness‐to‐pay.

To evaluate the robustness of our findings, we conducted probabilistic sensitivity analyses using 100,000 Monte Carlo simulations. When comparing the CBZ with HLA‐A*31:01 and CBZ without HLA‐A*31:01 screening strategies, the ICER demonstrated favorable results, with the CBZ with HLA‐A*31:01 screening strategy showing an acceptable ICER of 97.0% (Figure 2).

**Figure 3:**
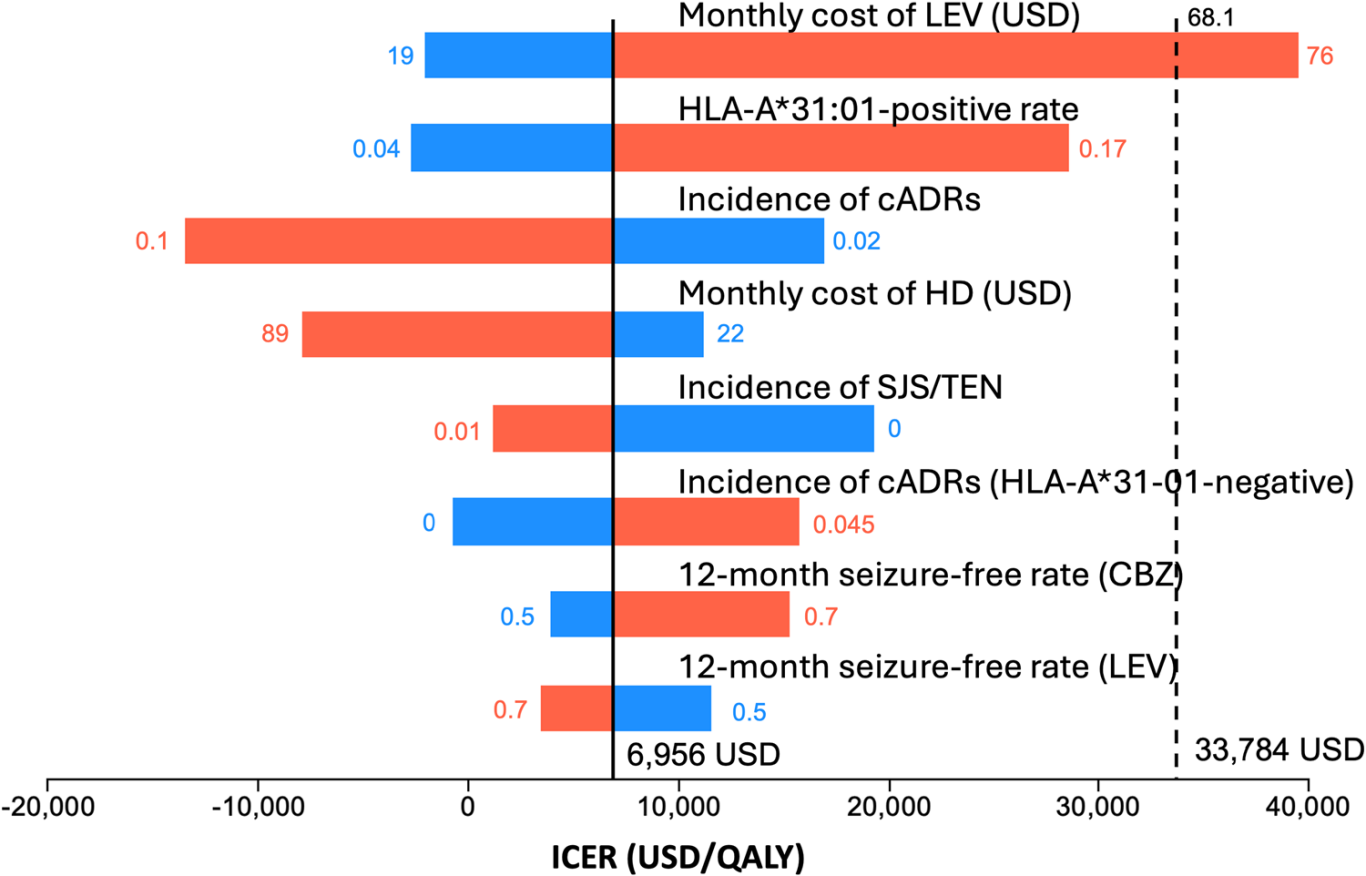
One‐Way Sensitivity Analysis; Tornado plot. The five parameters are most sensitive of all parameters. The numbers next to the bars indicate the ranges of the parameter in the sensitivity analysis. The red bars and numbers are higher parameters than baseline, and the blue bars are lower parameters. cADRs, cutaneous adverse drug reactions; CBZ, carbamazepine; ICER, incremental cost‐effectiveness ratios; LEV, levetiracetam; SJS, Stevens–Johnson syndrome; TEN, toxic epidermal necrolysis; WTP, willingness‐to‐pay.

One‐way sensitivity analysis was conducted on eight of the most sensitive parameters. When the monthly cost of LEV exceeded 68.1 USD, the ICER surpassed the WTP threshold. No other parameters caused the ICER to exceed the WTP threshold. Paradoxically, the results of the analysis demonstrated that an increase in the HLA‐A*31:01 positivity rate was associated with an upward trend in the ICER (Figure 3).

## DISCUSSION

Our cost‐effectiveness analysis demonstrated that implementing HLA‐A*31:01 screening before CBZ administration for newly diagnosed focal epilepsy is economically advantageous in the Japanese population. The ICER was calculated at 6956 USD/QALY gained, which is substantially below the WTP threshold of 33,784 USD.

The robustness of our results is supported by probabilistic sensitivity analysis, where the screening strategy maintained favorable cost‐effectiveness in 97.0% of simulations. A one‐way sensitivity analysis revealed that the monthly cost of LEV was the most influential parameter, with the ICER exceeding the WTP threshold only when LEV costs surpassed 68.1 USD. This finding has practical implications for prescription patterns, suggesting that the cost‐effectiveness of the screening strategy is maintained with standard or low‐dose regimens of LEV. Importantly, variations in other key parameters continued to yield ICERs below the WTP threshold.

A notable observation from our analysis is the paradoxical relationship between the HLA‐A31:01 prevalence and total costs (Figure 3). As the HLA‐A31:01 prevalence increases, total costs also rise. This occurs because in populations with a higher HLA‐A31:01 prevalence, fewer patients can use the less expensive CBZ, thereby requiring the use of costlier alternative ASMs such as LEV. Within the expected positive rate range in Japan, the ICER remains below the WTP threshold. However, some countries such as Algeria (Africa) and Colombia (South America) report HLA‐A31:01 prevalence rates of 30%–40% (Allele Frequency Net Database; https://www.allelefrequencies.net/), which are among the highest globally. It would be ethically problematic to conclude that genetic screening is less recommended in such high‐prevalence populations, as this approach would expose more individuals to potentially life‐threatening adverse reactions. This finding highlights the tension between economic analyses and ethical imperatives in medical decision‐making. The primary goal of pharmacogenetic screening is to prevent serious adverse events, and this protective benefit must be considered alongside economic factors.

Our results align with previous cost‐effectiveness analyses of HLA screening across various populations. Plumpton et al.^23^ found that pretreatment HLA‐A31:01 screening for CBZ therapy was cost‐effective in European populations, despite a low prevalence of approximately 2%–5%. Similarly, studies evaluating HLA‐B15:02 screening in various Asian populations have consistently demonstrated favorable cost‐effectiveness profiles, leading to incorporation into clinical practice guidelines. Furthermore, our previous analysis found that pretreatment HLA‐B*59:01 screening for clozapine was cost‐effective and beneficial for reducing the incidences of neutropenia and agranulocytosis in the Japanese population.^24^ Collectively, in Japan, these findings suggest that personalized medicine approaches incorporating genetic screening are clinically beneficial and economically sound across different healthcare systems and genetic backgrounds.

However, despite its demonstrated cost‐effectiveness, the implementation of personalized medicine approaches incorporating genetic screening, such as that for HLA‐A*31:01, into clinical practice faces several challenges in Japan. Currently, the Japanese health insurance system does not cover HLA allele testing for pre‐prescription screening, requiring patients or healthcare institutions to bear the full cost. This economic barrier, coupled with limited awareness among healthcare professionals regarding the importance of pharmacogenetic screening, hinders its widespread adoption. Our findings provide compelling evidence to support policy changes incorporating genetic screening in the Japanese healthcare insurance system, potentially reducing the incidence of serious adverse drug reactions while maintaining cost‐effectiveness.

This study has several limitations. First, our model focused specifically on cutaneous adverse drug reactions and did not incorporate long‐term metabolic adverse effects of CBZ, such as cardiovascular events, osteoporosis, and dyslipidemia, caused by enzyme induction. While this represents a limitation in comprehensively evaluating CBZ therapy costs, HLA‐A*31:01 screening specifically targets cutaneous adverse reactions and has no established association with metabolic adverse effects. Since these long‐term metabolic adverse effects would occur equally in both screening and non‐screening strategies, their inclusion would not alter the relative cost‐effectiveness of HLA screening, which is the primary focus of our analysis.

Second, our model assumed no treatment discontinuation after the first 12 months for patients who remained on therapy. However, this assumption reflects real‐world clinical patterns, as most treatment‐related discontinuations due to adverse effects occur within the first year of antiseizure medication therapy, and patients who tolerate treatment through this period typically continue long‐term without discontinuation. This assumption is therefore considered clinically reasonable for our long‐term cost‐effectiveness projection.

Third, treatment strategies for drug‐resistant epilepsy are complex and highly individualized. While this study restricted alternative medications to lacosamide, topiramate, zonisamide, and lamotrigine, other agents, including brivaracetam, valproic acid, phenobarbital, and phenytoin, are commonly used in clinical practice.

While we used normal distributions for utility values rather than beta distributions typically recommended for bounded parameters, our sensitivity analyses demonstrated that the ICER remained robust and far below the willingness‐to‐pay threshold across the entire range tested.

Finally, while CBZ is also used in the treatment of bipolar disorder and trigeminal neuralgia, HLA allele screening for these conditions has not been evaluated. Further analyses are necessary to generalize the present findings across all potential applications.

## CONCLUSION

The results of the present study indicate that HLA‐A*31:01 screening before CBZ treatment is cost‐effective in the Japanese population. These findings support incorporating genetic screening into Japan's healthcare coverage system to enable more personalized medicine approaches in epilepsy treatment.

## AUTHOR CONTRIBUTIONS

Yasushi Maruyama, Kohei Ninomiya, Hiroki Kimura, and Masashi Ikeda contributed to the conception and design of the study. Yasushi Maruyama, Kohei Ninomiya, Shiori Ogawa, Hiroki Kimura, Takuji Nishida, and Masashi Ikeda provided substantial contributions to the analysis and interpretation of the clinical data. Yasushi Maruyama and Shiori Ogawa wrote the first draft. All authors contributed to and approved the final version of the manuscript for submission.

## CONFLICT OF INTEREST STATEMENT

Masashi Ikeda has received speaker's honoraria from Meiji Seika Pharma, Otsuka Pharmaceutical, Takeda Pharmaceuticals, Lundbeck Japan KK, Mochida Pharmaceutical, Jansen Pharmaceutica KK, Tanabe Mitsubishi Pharma, MSD, Eisai, Kyowa Pharma Chemical, Towa Pharmaceutical, and Viatris. Takuji Nishida has received speaker's honoraria from UCB Japan, Eisai, Daiichi Sankyo, and Sumitomo Pharma. The remaining authors declare no conflicts of interest.

## ETHICS APPROVAL STATEMENT

This study was a cost‐effectiveness modeling analysis using previously published data and did not involve human subjects. Therefore, ethics committee approval was not required.

## PATIENT CONSENT STATEMENT

N/A.

## CLINICAL TRIAL REGISTRATION

N/A.

## Data Availability

The data that support the findings of this study are available from the corresponding author upon reasonable request.
